# STAS Domain Only Proteins in Bacterial Gene Regulation

**DOI:** 10.3389/fcimb.2021.679982

**Published:** 2021-06-21

**Authors:** Brian E. Moy, J. Seshu

**Affiliations:** South Texas Center for Emerging Infectious Diseases (STCEID), Department of Biology, The University of Texas at San Antonio, San Antonio, TX, United States

**Keywords:** STAS domain_1_, Pfam01740_2_, sulfate transport anti-sigma antagonist_3_, STAS Domain only Proteins_4_, bacterial gene regulation_5_, anti-sigma antagonist_6_, sigma factor_7_

## Abstract

Sulfate Transport Anti-Sigma antagonist domains (Pfam01740) are found in all branches of life, from eubacteria to mammals, as a conserved fold encoded by highly divergent amino acid sequences. These domains are present as part of larger SLC26/SulP anion transporters, where the STAS domain is associated with transmembrane anchoring of the larger multidomain protein. Here, we focus on STAS Domain only Proteins (SDoPs) in eubacteria, initially described as part of the *Bacillus subtilis* Regulation of Sigma B (RSB) regulatory system. Since their description in *B. subtilis*, SDoPs have been described to be involved in the regulation of sigma factors, through partner-switching mechanisms in various bacteria such as: *Mycobacterium. tuberculosis, Listeria. monocytogenes, Vibrio. fischeri, Bordetella bronchiseptica*, among others. In addition to playing a canonical role in partner-switching with an anti-sigma factor to affect the availability of a sigma factor, several eubacterial SDoPs show additional regulatory roles compared to the original RSB system of *B. subtilis.* This is of great interest as these proteins are highly conserved, and often involved in altering gene expression in response to changes in environmental conditions. For many of the bacteria we will examine in this review, the ability to sense environmental changes and alter gene expression accordingly is critical for survival and colonization of susceptible hosts.

## Introduction

Sulfate Transport Anti-Sigma antagonist (STAS) domains (Pfam01740) can be found in all branches of life, from eubacteria to mammals, as a conserved fold encoded by highly divergent amino acid sequences (Aravind and Koonin, 2000). STAS domains are found as a single domain within small, STAS Domain only Proteins (SDoPs) such as RsbV of *B. subtilis* and within larger, multidomain proteins such as SulP of *Escherichia. coli* or SLC26 family of transporters in mammals. Our *in-silico* analysis revealed the presence of SDoPs in all pathogenic bacterial species examined, however it is possible that bacterial species may exist that lack an SDoP. Canonical SDoPs in *B. subtilis* have been shown to be involved in the regulation of sigma factors that bind RNA polymerase to affect transcription (Kang et al., 1996; Campbell et al., 2008). Here, we review the role of canonical and non-canonical SDoPs present in *B. subtilis* and in different species of bacteria, where they play a wide variety of roles in regulating the expression of genes in response to an array of external stimuli.

These SDoPs and the STAS domains within SulP/SLC26 anion transporters have a conserved structure consisting of 4 α-helices and 5 β-sheets, with a highly conserved loop between the 3rd α-helix and 2nd β-sheet (Aravind and Koonin, 2000). It has been shown that *B. subtilis* SDoP SPOIIAA binds GTP (and to a lesser extent ATP), and possesses weak NTPase activity that is reduced by phosphorylation, or by mutation of serine residue 58 to alanine in the conserved loop (Najafi et al., 1996; Masuda et al., 2004). It has been suggested that the conserved loop is involved in phosphate binding, with the downstream β-sheet being involved in the accommodation of the rest of the NTP molecule (Aravind and Koonin, 2000).

Canonically, these SDoPs positively regulate sigma factors by interacting with their cognate anti-sigma factor, which has protein kinase activity. This anti-sigma factor then phosphorylates the SDoP on a conserved serine residue in the conserved loop, inactivating it. The inactivated (phosphorylated) SDoP then dissociates from the anti-sigma factor, allowing it to sequester its cognate sigma factor. The SDoP can later be reactivated by an input phosphatase, leading to dephosphorylation of the conserved serine residue and interactions between the SDoP and the anti-sigma factor (Aravind and Koonin, 2000).

## STAS Domains as Part of SLC26/SulP Family

Multidomain STAS domain containing proteins encoded by the genomes of *E. coli*, and *M. tuberculosis* are members of the SLC26/SulP family and are involved in various biological functions, such as transporting ions and carboxylic acids (Alper and Sharma, 2013). Additionally, there are examples of STAS domains within larger multidomain proteins that are not in the SLC26/SulP family, such as *all4160* in *Anabaena*, which encodes a glycosyltransferase and an N-terminal STAS domain (Wang et al., 2007). SLC26/SulP anion transporters contain an integral membrane domain with two inverted repeats of seven transmembrane domains and a cytoplasmic STAS domain (Compton et al., 2011; Compton et al., 2014; Geertsma et al., 2015). The role of the STAS domain in these SLC26/SulP has not been fully explored, but it has been suggested to be important for targeting proteins to the membrane (Sharma et al., 2011; Compton et al., 2014). However, recent work has demonstrated that the STAS domain does not play a direct role in protein targeting in bacteria, but instead is required for protein stabilization and functionality (Birke and Javelle, 2016; Lolli et al., 2016). In mammals, such as humans, SLC26 transporters play a diverse and critical role as anion exchangers, with three known mutations that can lead to early onset hereditary diseases: chondrodysplasias, chloride diarrhea, and deafness (Compton et al., 2011; Alper and Sharma, 2013; Compton et al., 2014). While their relevance to human disease has led to these SLC26 transporters and their component STAS domain being the subject of intensive study, there remains a lack of information on the role of SDoPs in bacterial physiology and gene regulation.

## Canonical STAS Domain Only Proteins

**Figure 1:**
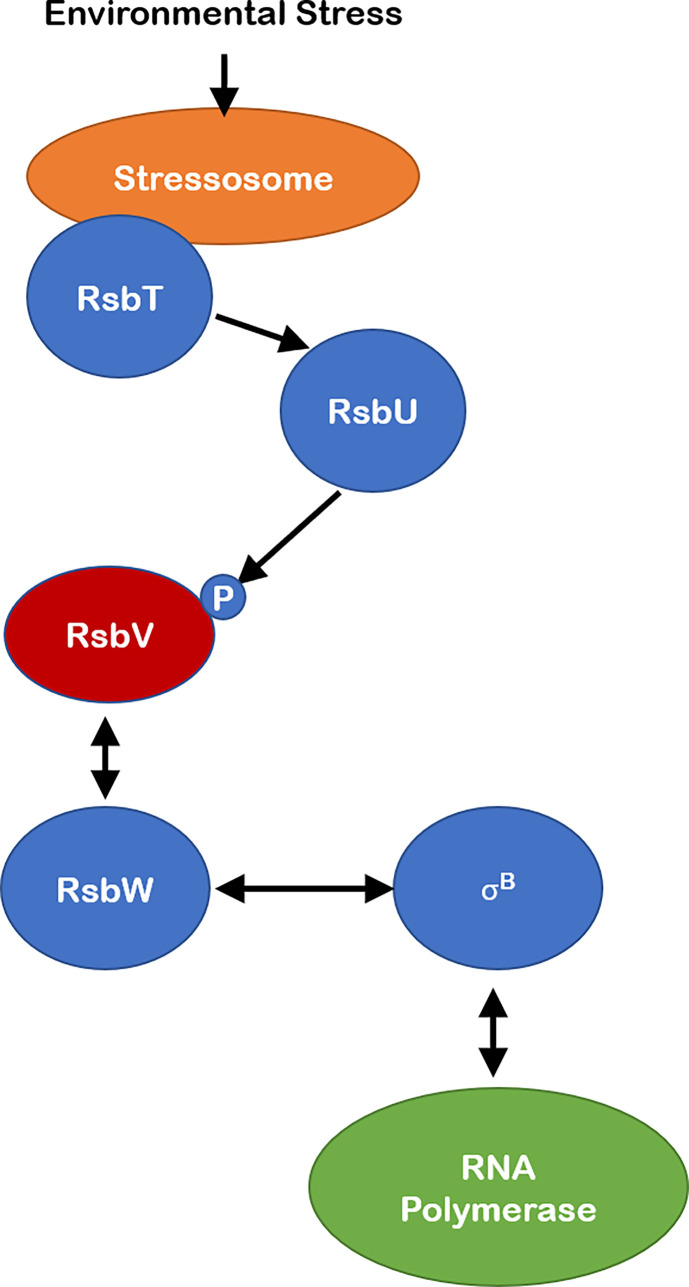
Schematic of canonical SDoP in the Regulation of Sigma B pathway in *B subtilis*. Environmental stress leads to activation of the stressosome, allowing for activation of the RsbT phosphatase activator. This allows RsbU phosphoserine phosphatase to dephosphorylate a conserved serine residue on SDoP – RsbV- which in its unphosphorylated state interacts with anti-sigma factor RsbW, freeing σ^B^ to bind the core RNA polymerase and initiate transcription of genes in the general stress response operon. The anti-sigma factor RsbW possesses serine kinase activity such that when the environmental stress is no longer present RsbV is phosphorylated, leading to its dissociation from RsbW. RsbW then sequesters σ^B^, leading to decreased expression of its operon.

Perhaps the best studied example of SDoPs in bacteria is in the regulation of sporulation in *B. subtilis* (**Figure 1**). Specifically, these proteins regulate gene expression by indirectly affecting the ability of the alternative sigma factor, σ^B^, to bind the core RNA polymerase (Dufour and Haldenwang, 1994; Rodriguez Ayala et al., 2020). Generally, these systems can be described as phosphorylation-dependent partner-switching regulatory systems involving anti-sigma factors/serine kinases, serine-threonine phosphatases, and anti-sigma antagonists (Rodriguez Ayala et al., 2020). The Regulation of σ^B^ (Rsb) system is an example of such partner-switching regulatory systems involved in the rapid initiation of transcription of more than 150 general stress proteins following exposure to environmental stress in *B. subtilis* (Haldenwang and Losick, 1979; Binnie et al., 1986; Hecker et al., 2007; Losick and Pero, 2018). In a normal, unstressed state the anti-sigma factor RsbW binds to σ^B^ and maintains it in an inactive state (**Figure 1**). Environmental stress leads to the activation of an input phosphatase (e.g., RsbU), which dephosphorylates the SDoP RsbV, at a conserved serine residue. Dephosphorylated RsbV can then bind to and sequester the anti-sigma factor RsbW, freeing σ^B^ to bind to the core RNA polymerase and initiate transcription of general stress proteins (Benson and Haldenwang, 1993; Alper et al., 1996; Price, 2001). RsbW also has serine kinase activity which phosphorylates RsbV, such that when the input phosphatase (triggered by environmental/energy stress) is no longer active, RsbV remains phosphorylated. RsbW then dissociates from phosphorylated RsbV, allowing RsbW to sequester σ^B^ until another environmental stress event is encountered (**Figure 1**) (Benson and Haldenwang, 1993; Alper et al., 1996; Kang et al., 1996; Yang et al., 1996; Rodriguez Ayala et al., 2020).

SpoIIAA, another SDoP in *B. subtilis*, also plays a role in controlling sporulation-related genes by regulating σ^F^ *via* a partner-switching mechanism with an anti-sigma factor, similar to the regulation of σB system described above. Under non-spore forming conditions σ^F^ is sequestered by the anti-sigma factor SpoIIAB. Following exposure to spore forming conditions (e.g., nutrient starvation), SpoIIAA is activated by dephosphorylation of a conserved serine residue by SpoIIE, leading to interactions between SpoIIAA and SpoIIAB. This interaction between SpoIIAA and SpoIIAB frees σ^F^ to initiate transcription of sporulation genes (Losick and Stragier, 1992; Duncan et al., 1996; Piggot and Hilbert, 2004; Pedrido et al., 2013). In addition to partner-switching with SpoIIAB, SpoIIAA also has been shown to serve as a negative regulator of Spo0A activation. This results in a negative feedback loop that tightly controls regulation of sporulation genes, as transcription of SpoIIAA, SpoIIAB and σ^F^ are regulated by Spo0A (Arabolaza et al., 2003). This negative feedback loop contributes to blocking the expression of Spo0A-dependent genes (SpoIIAA, SpoIIAB, σ^F^), whose products are no longer needed and are likely to contribute to proper cell-specific activation of σ^F^ and σ^E^ (Arabolaza et al., 2003).

While this phosphorylation-dependent partner-switching regulatory system between an anti-sigma antagonist, an anti-sigma factor/serine kinase, and a sigma factor was initially described in *B. subtilis*, it has since been described in pathogenic Gram-positive bacteria such as *Staphylococcus aureus* (Ziebandt et al., 2001; Jonsson et al., 2004) and *M. tuberculosis* (Beaucher et al., 2002; Parida et al., 2005). Additionally, there are examples of similar partner-switching regulatory systems containing an SDoP in pathogenic gram-negative bacteria such as *B. bronchiseptica* (Mattoo et al, 2004), *Chlamydia trachomatis* (Mattoo et al., 2004; Hua et al., 2006), symbionts like *Vibrio fischeri* (Thompson and Visick, 2015), and in environmental bacteria like *Shewanella oneidensis* (Bouillet et al., 2016). In these systems, the SDoPs show distinct roles in regulating pathways involved in 1) Type III secretion and virulence (Mattoo et al., 2004; Kozak et al., 2005), 2) biofilm formation (Thompson and Visick, 2015) and 3) growth and stress adaptation (Thompson et al., 2015; Bouillet et al., 2016).

*Listeria monocytogen*es, an enteric pathogen, utilizes a system like the regulation of σ^B^ in *B. subtilis* in regulating its response to acid stress, and impacting its ability to establish infection in the gastrointestinal tract. *L. monocytogenes* encodes *rsbR, rsbS, rsbT, rsbU, rsbV, rsbW, rsbX* and σ^B^. Briefly, upon environmental stress RsbT is released from the stressosome and initiates a signal cascade by associating with serine phosphatase RsbU, which then dephosphorylates a conserved serine residue on SDoP, RsbV. The anti-sigma factor RsbW, which normally sequesters σ^B^, binds dephosphorylated RsbV with a higher affinity than RsbW, freeing σ^B^ to initiate transcription of its operon. This complex regulatory system contributes to survival of *L. monocytogenes*, both inside and outside the host, by affecting its virulence and survival under environmental stress, respectively (Chaturongakul and Boor, 2004; Chaturongakul and Boor, 2006; Shin et al., 2010; Utratna et al., 2014; O’Donoghue et al., 2016; Guerreiro et al., 2020a; Guerreiro et al., 2020b; Hsu et al., 2020).

In *M. tuberculosis*, a partner-switching system has been described that controls the availability of σ^F^ and is referred to as the Regulation of Sigma F (Rsf) system. In this system two separate SDoPs, RsfA and RsfB, are involved in a partner-switching system; with RsfB showing higher homology to RsbV of *B. subtilis*. This allows for a level of functional redundancy in the mycobacterial regulatory circuit and could allow for the bacterium to readily adapt to a wide range of conditions requiring differential activation of σ^F^ (Beaucher et al., 2002). RsfA-like proteins are present only in mycobacterial species that cause tuberculosis, suggesting a possible role for these proteins in virulence (Beaucher et al., 2002; Manganelli et al., 2004). In *M. smegmatis*, RsfA has been shown to interact more strongly with RsbW1 than RsfB (Singh et al., 2015), and acts as a stronger anti-σ^F^ antagonist than RsfB (Oh et al., 2020). A serine residue at position 63 of RsfB of *M. smegmatis*, and its phosphorylation by RsbW2, determines the functionality of RsfB as an anti-sigma antagonist. Furthermore, RsfB was identified as the major anti-σ^F^ antagonist in *M. smegmatis* (Oh et al., 2020).

Similarly, a partner-switching system containing components like those involved in the regulation of σ^B^ system in Bacillus have been reported in *Chlamydia trachomatis*. The chlamydial partner-switching system includes two SDoPs; RsbV1 and RsbV2 (Mattoo et al., 2004), a homolog of the anti-sigma antagonist RsbWCt, and the phosphatase RsbUCt (Hua et al., 2006). However, unlike the alternate sigma factor σ^B^ which is regulated in *Bacillus*, the housekeeping sigma factor σ^66^ is controlled by this partner-switching regulatory system *in C. trachomatis* (Hua et al., 2006; Thompson et al., 2015). It was found that RsbWCt interacts with unphosphorylated RsbV1 and RsbV2, with a preference for RsbV1. Additionally, RsbUCt exhibited *in vitro* phosphatase activity for RsbV1 but not RsbV2 (Thompson et al., 2015). These findings led to a model of the Rsb regulatory system in *C. trachomatis*: under steady-state conditions the expression level of RsbUCt, RsbV1, and RsbWCt provide an equilibrium in which σ^66^ availability is high, facilitating normal growth and development. An accumulation of non-phosphorylated RsbV1, due to increased expression/activity of the phosphatase RsbUCt, facilitates RsbV1 binding to RsbWCt with a concomitant increase in the levels of σ^66^ (Thompson et al., 2015). The current model suggests that increased levels of alpha-ketoglutarate encountered in the host cell leads to increased RsbUCt mediated phosphatase activity on RsbV1, leading to dissociation of RsbWCt from its target protein to re-phosphorylate RsbV1, allowing the target protein to affect activation of the TCA cycle (Soules et al., 2020).

Another example of a bacterial SDoP has been described in *Pseudomonas aeruginosa*, where the RsbV homolog PA3347 participates in a partner-switching regulatory system to affect expression of genes involved in swarming (Hsu et al., 2008; Bhuwan et al., 2012). In this system, it has been shown that PA3347 is phosphorylated at serine 56 by its upstream neighbour, PA3346 response regulator, which has both phosphatase and kinase activities. In *P. aeruginosa*, the histidine-containing phosphotransfer-B (HptB) is involved in transferring phosphoryl groups from multiple sensor kinases to PA3346, which in turn controls flagellar gene expression by interactions with PA3347 (Bhuwan et al., 2012). When PA3347 (STAS domain) is dephosphorylated by PA3346, it binds to the anti-sigma factor FlgM, allowing σ^28^ to dissociate from FlgM and bind to the core RNA polymerase, form an active holoenzyme, and initiate transcription of flagellar genes. Interestingly, it was also demonstrated that PA3346 can phosphorylate PA3347, which in turn activates the C-terminal region of PA3346 to act as an anti-σ factor. These events lead to inactivation of an unidentified σ factor, ultimately affecting transcription of genes involved in swarming motility (Bhuwan et al., 2012).

## Non-Canonical SDoPs

**Figure 2:**
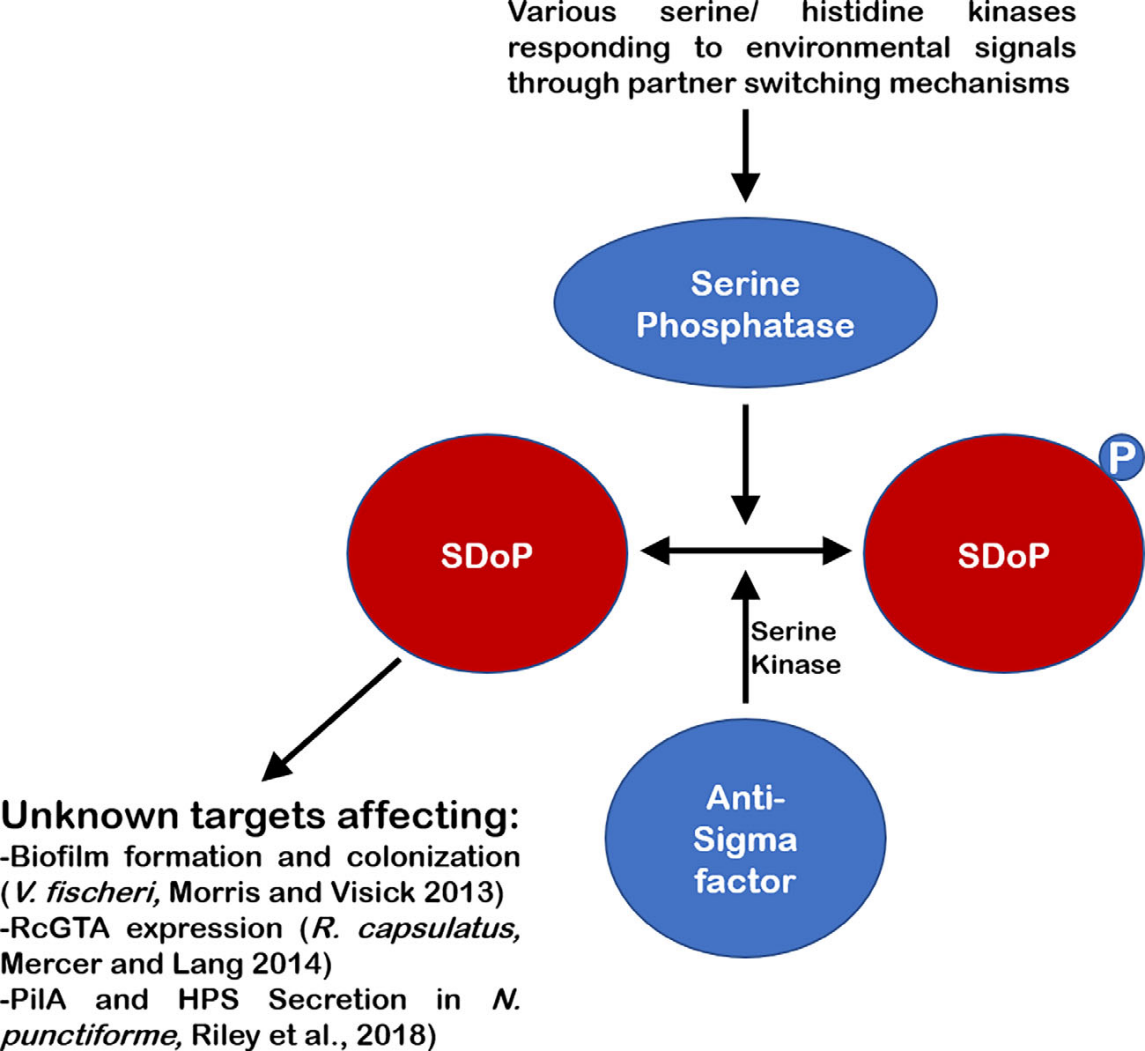
Schematic of several non-canonical SDoPs. Environmental signals lead to activation of various partner-switching mechanisms, leading to the activation of a serine phosphatase which can dephosphorylate the SDoP at a conserved serine residue. This dephosphorylation allows the SDoP to affect gene expression through various unknown targets to produce various phenotypes in different bacteria. These systems also generally contain an anti-Sigma factor which has serine kinase activity and phosphorylates the SDoP, inactivating it, when the signal leading to activation of the pathway is no longer present.

While the canonical systems with a SDoP, whose phosphorylation at a conserved serine residue leads to dissociation from an anti-sigma factor/serine kinase (**Figure 1**); there are several examples of SDoPs that appear to be active in their unphosphorylated form, interacting with unknown partners that may include sigma factors (**Figure 2**). One such example is in the *Vibrio fischeri* symbiosis polysaccharide (*syp*) locus, where unphosphorylated SDoP SypA is active and serves as the output to initiate transcription of genes involved in biofilm formation (Morris and Visick, 2010; Morris et al., 2011; Morris and Visick, 2013a). In this system, unphosphorylated SypA acts upon an unknown target to promote biofilm formation (Morris and Visick, 2013a; Thompson and Visick, 2015; Thompson and Visick, 2017). In non-biofilm forming conditions, the dual serine kinase/phosphatase SypE phosphorylates SypA at serine 56 and prevents it from activating biofilm genes (Sievers et al., 2011; Thompson and Visick, 2015; Thompson and Visick, 2017). This is like a Serine 55 residue in *B. bronchiseptica* SDoP BtrV, which plays a key role in mediating interactions with BtrW, where phosphorylation of this conserved serine residue promotes binding to BtrW (Kozak et al., 2005), as opposed to inhibiting binding as seen in other canonical SDoPs described above.

As mentioned above, in *B. bronchiseptica* the phosphorylation of an SDoP, BtrV, has been shown to promote binding to the anti-sigma factor BtrW (Kozak et al., 2005), as opposed to this phosphorylation in canonical systems leading to dissociation of the SDoP from the anti-sigma factor. This partner-switching mechanism between BtrV and BtrW has been shown to play a role in regulation of the Type III secretion system, which plays a critical role in the colonization of a host by *B. bronchiseptica* (Mattoo et al., 2004; Kozak et al., 2005). While BtrU, BtrV and BtrW show homology to the Rsb system of *Bacillus*, the *Bordetella* system is different in that all three of these are required for T3SS secretion and none act as a negative regulator. Evidence suggests that BtrV exerts post-transcriptional control required for translation, while BtrU and BtrW are involved in regulation of the secretion process (Mattoo et al., 2004; Kozak et al., 2005; Ahuja et al., 2016; Kamanova, 2020).

In *Rhodobacter capsulatis*, the SDoP RbaV serves as the output of a regulatory system to affect expression of genes involved in motility (Mercer and Lang, 2014). In this system, unphosphorylated RbaV works as the output of the system to affect expression of an unknown target, consequently leading to increased expression of horizontal gene transfer agents (Mercer and Lang, 2014). Like previously described systems, RbaV has conserved serine residues at positions 56 and 57, and it is suggested that phosphorylation of one of these residues leads to activation/deactivation of RbaV (Mercer and Lang, 2014). It is unclear if the contributions of the anti-sigma factor RbaW by itself, or the interactions between RbaV-RbaW, mediates the control of a cognate σ factor. Alternatively, it remains to be determined if RbaV and its partner RbaW are directly affecting gene expression through a partner-switching mechanism.

An additional example of a non-canonical SDoP has been described in the *hmp* (hormogonium motility and polysaccharide) locus of the filamentous cyanobacterium *Nostoc punctiforme* (Riley et al., 2018). In this partner-switching regulatory system, it is suggested that HmpV is active when dephosphorylated by HmpU, and inactive when phosphorylated by anti-sigma factor HmpW (Riley et al., 2018). Currently, it is unknown whether unphosphorylated HmpV acts directly on an unknown target, or if it interacts with another protein to produce the observed regulatory effects.

## Summary

Partner-switching mechanisms utilizing SDoPs were originally described in *B. subtilis*, as a regulatory system to control the availability of sigma factors in response to environmental stress (Losick and Stragier, 1992; Kang et al., 1996; Rodriguez Ayala et al., 2020). In addition to the control of sigma factors in *B. subtilis*, these SDoPs have been shown to regulate survival of *L. monocytogenes* inside the host and contribute to the virulence of this pathogen (Chaturongakul and Boor, 2004). Moreover, a partner-switching regulatory system containing a SDoP controlling the Type III secretion in *B. bronchiseptica* adds to the significance of this class of proteins in regulating bacterial pathogenesis (Mattoo et al., 2004; Kozak et al., 2005). These SDoPs also contribute to the survival of *M. tuberculosis* and *C. trachomatis* within host cells (Beaucher et al., 2002; Thompson et al., 2015). In the above examples, the regulatory effects have been at least partially attributed to partner-switching of the SDoPs with an anti-sigma factor, freeing a sigma factor to initiate transcription of its respective operon. However, even in these well studied systems there remain questions about additional roles for these SDoPs in gene regulation in response to external stimuli, which is often intrinsically tied to survival in a host in several of the species examined here.

As shown in **Figure 2**, SDoPs are also known to directly influence gene expression through a variety of mechanisms as in *V. fischeri*, *R. capsulatis* and *N. punctiforme* (Bhuwan et al., 2012; Morris and Visick, 2013b; Mercer and Lang, 2014; Riley et al., 2018). These examples highlight the multiple roles that these SDoPs play in responding to external stimuli and warrants further research into these ubiquitous regulatory proteins.

Here, we specifically focused on SDoPs, in comparison to the larger SLC26/SulP superfamily of proteins which have been characterized in greater detail. In many bacteria the components of the partner-switching mechanisms mediated by SDoPs show a high degree of homology in structure and function to the canonical systems initially described in *B. subtilis* (Dufour and Haldenwang, 1994) (**Figure 1**). However, there are examples of non-canonical systems where this unique class of proteins interacts with unknown targets to influence the physiology and survival of bacteria within or outside of a host (**Figure 2**). Moreover, several other bacterial species encode for homologs of SDoPs, although their contribution to the pathophysiology of these prokaryotes are yet to be established. In some cases, SDoPs are part of operons involved in motility or chemotaxis with a possibility of regulating sub-global gene expression levels specific to the operon or exhibit a widespread global transcriptional effect. It is also unknown if SDoPs as a class of proteins can complement their functions in heterologous bacterial systems, or if these SDoP confer narrow, organism-specific regulatory effects consistent with the pathophysiology of the prokaryote. A greater understanding of the role of SDoPs may unravel novel global or sub-global regulatory networks critical in regulating bacterial adaptation and survival in response to changes in their environments.

## Author Contributions

BEM conceived and wrote the review and JS edited the review. All authors contributed to the article and approved the submitted version.

## Funding

This work was partly supported by Public Health Service Grant AI123837 from the National Institute of Allergy and Infectious Diseases. Work in Seshu laboratory is generously supported by Bay Area Lyme Foundation and The Brown Foundation.

## Conflict of Interest

The authors declare that the research was conducted in the absence of any commercial or financial relationships that could be construed as a potential conflict of interest.
